# Incidence of early postoperative complications requiring surgical revision for recurrent lumbar disc herniation after spinal surgery: a retrospective observational study of 9,310 patients from the German Spine Register

**DOI:** 10.1186/s13037-018-0157-1

**Published:** 2018-05-21

**Authors:** Juan Manuel Vinas-Rios, Martin Sanchez-Aguilar, Fatima Azucena Medina Govea, Viktor Von Beeg-Moreno, Frerk Meyer, DWG Registry-group

**Affiliations:** 1 Sana Klinikum Offenbach, Starkenburgring 66, 63069 Offenbach, Germany; 2 Clinic Epidemiology, Universidad Autonoma de San Luis Potosi, San Luis Potosí, Mexico; 3 University Johann W. Goethe Faculty of Medicine, Frankfurt am Main, Germany; 4 University Clinic Evangelical Hospital Oldenburg, Oldenburg, Germany

**Keywords:** Disc herniation, Early recurrence, Lumbar spine, Spine Register

## Abstract

**Background:**

The recurrence rate in lumbar disc herniations (LDH) has been reported between 5 and 25%. There are only few data about this phenomenon that occurs within days of the initial operation. We analyse early recurrent LDH by analysis of data from the German Spine register.

**Methods:**

Data from patients undergoing disc herniation surgery in the lumbar region were extracted from the German Spine Registry between 1st January 2012 and 31st December 2016. Patients with early recurrent LDH within days of initial surgery were separately analysed.

**Results:**

A total of 9310 surgeries for LDH were documented in the German Spine Register. From these patients 115 (1.2%) presented an early recurrent disc surgeries within days of the initial surgery. The mean age was 70 ± 2.50 years. Most affected segment was L4/5 (47 cases, 41%), followed by L3/4 (45 cases, 39%). The most of our patients showed a normal or overweight Body Mass Index. Surgery for early recurrent LDH was associated with a high rate of incidental durotomies (20 cases, 17.6%). In 3 cases (2.6%) therapy with a lumbar drain was necessary.

**Conclusions:**

The rate of early recurrent LDH within days of surgery is 1.2%. Age seems to be an important factor in early recurrent LDH while obesity does not. The data of the German Spine Register seems to have a reliable data collection system that can perform multicentre data analysis. The databases from this Register could be used in the future for various purposes, such as the evaluation of multicentre surgical techniques, results in patients with various surgical procedures and basic research in spine surgery.

## Background

The recurrence rate in lumbar disc herniations (LDH) has been reported to be 5 to 25%. Nowadays there are only few data about this phenomenon that occur within days of the initial operation. The most affected segment is L4/5 [1–3]. On the other hand, there are few data available on the incidence of recurrence while the patient is still in hospital [4, 5].

Currently it is not clear which factors exert an influence on the genesis of this recurrency. Due to the biomechanics of the pathology, advanced age and pre-existing conditions such as overweight and obesity, as well as the localisation of the affected segment could play a role in the pathology [2, 6–8].

## Methods

The aim of this study is that with the help of the available data of the German Spine Register (DWG register), the question should be answered, how often an early recurrence can be expected during the inpatient stay after microsurgical Lumbar nucleotomy.

From 1st January 2012 to 31st December 2016, 9310 intervertebral disc operations were recorded using the DWG Register. These data were analysed for the occurrence of early recurrence and for any risk factors for this. With regard to all patients with lumbar disc herniation (*n* = 9310), 115 early recurrences were detected (1.2%).

## Results

We found 115 early recurrences (1.2%) in 9310 surgical procedures due to a lumbar disc herniation.

The mean age of patients with an early recurrence was 70 ± 2.50 years with 75 women and 50 men.

The segment L4 / 5 (*n* = 47, 41%) was most frequently affected, followed by the segment L3 / 4 (*n* = 45, 39%) and the segment L5 / S1 (*n* = 23, 20%).

Regarding Body Mass Index (BMI): 4 patients (3.5%) were underweight (< 20 BMI), 45 (39%) were of normal weight (20–25 BMI), 45 (39%) patients were overweight (26–30 BMI), 19 (16.8%) had Grade 1 obesity (31–35 BMI) and 2 (1.7%) had Grade 2 obesity (> 35 BMI).

A dura-injury occurred in *n* = 20 (17.6%), while *n* = 3 (2.6%) had to be treated with a lumbar drainage system.

Of the patients treated for a herniated disc (*n* = 84, 82%), 18 (17%) experienced an improvement in their symptoms, while none (0.9%) experienced no relief.

For a detailed view of the overall results, see Table 1.

**Table 1:** Demographics and clinical variability of examined patients with recurrent herniated disc after a lumbar disectomy

	Early Lumbar Disc Herniation recurrence
Patients	115/9310 (1.2%)
Gender (m/f)	50/75
Age (years) (Median, range)	70 ± 2.50
Body Mass Index (BMI)	
< 20 (Underweight)	4 (3.5%)
20–25 (Normal weight)	45 (39%)
26–30 (Overweight)	45 (39%)
31–35 (Obesity grade 1)	19 (16.8%)
> 35 (Obesity grade 2)	2 (1.7%)
Affected segment	
L3/L4	45 (39%)
L4/L5	47 (41%)
L5/S1	23 (20%)
Intraoperative complications	
Dura-injury	20 (17.6%)
Postoperative complications	
CSF Leakage	3 (2.6%)
Symptoms relief	
1. Complete	84 (82%)
2. Partial	18 (17%)
3. None	1 (0.09%)

## Discussion

This analysis of data from the DWG Register from 9310 patients shows an early recurrence rate of 1.2%. The L4 / 5 segment is most frequently affected. Misleadingly, one might come to the premature conclusion that the most frequently affected segment would be L5 / S1 [2], as it represents biomechanically represents the location of maximum lumbar region mobility. Considering our results and the data presented in the literature, it would be useful if spinal biomechanics were more closely examined in terms of the pathogenesis of recurrences in future studies [2].

Due to the loss of elasticity of the tissue, which can create conditions for a primary prolapse, age is a very important factor in the pathogenesis of this pathology [6, 8]. This is also confirmed by the data we obtained in our study. However, obesity may not be a factor in the development of early relapse, as this pathology may not be due to the biomechanics of the L4 / L5 segment, since axial load over the intervertebral space may be of little concern for recurrence [2, 7].

**Fig. 1:**
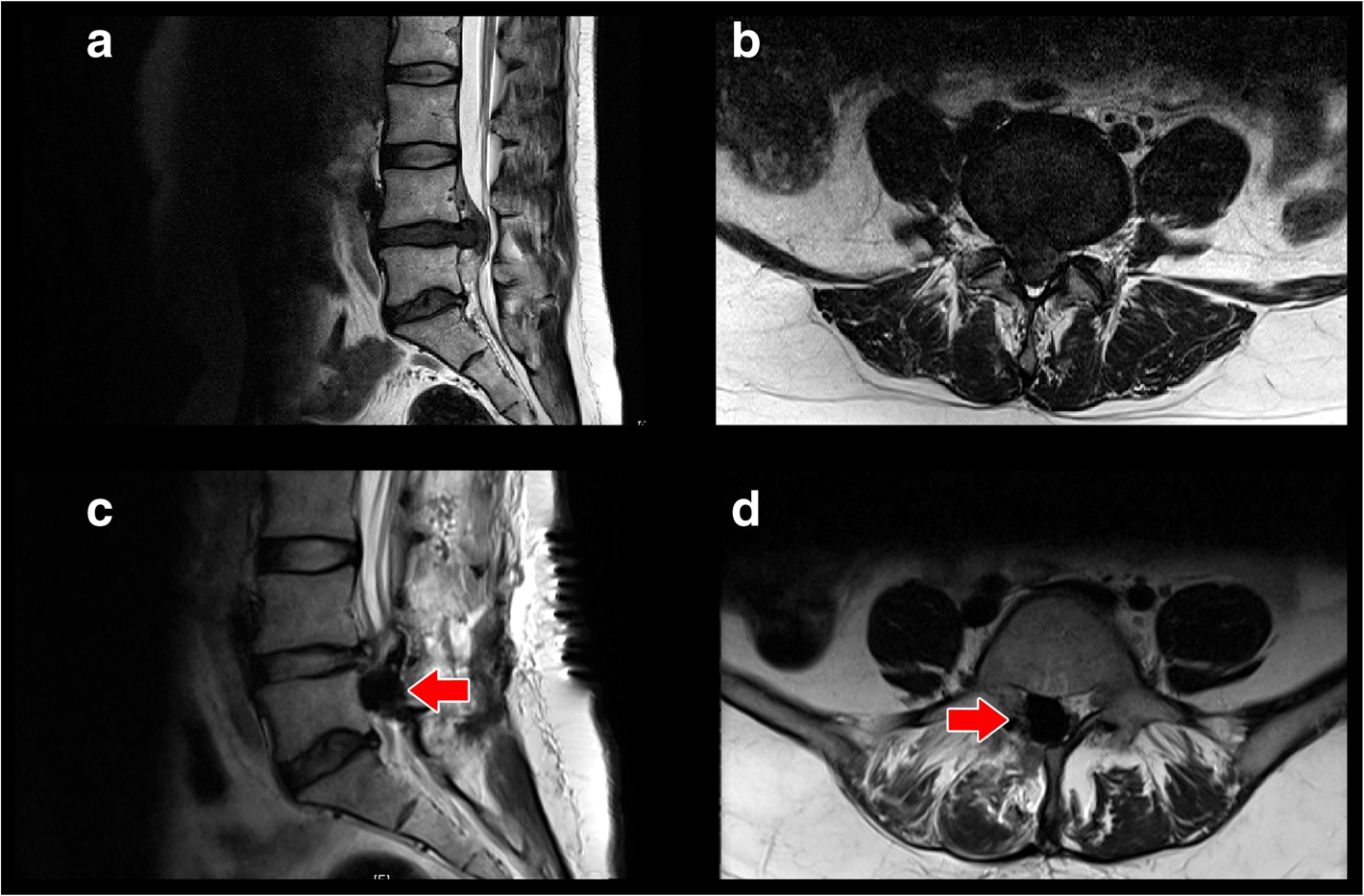
MRI T2 weight from the Lumbar spine. **a** and **b** show a massive Lumbar Disc Herniation in the Segment L4/L5 in sagital and axial views. **c** and **d** show after 36 h an early recurrent disc herniation with postoperative Bleeding (↑) and compression of the Cauda Equina causing bowel and bladder retention in sagital and axial views

Occasionally, as a result of the enormous compression of the epidural veins in to the spinal canal through the Lumbar Disc Herniation, significant postoperative bleeding with considerable compression of the neural structures as well as an early Lumbar Disc Herniation recurrence may occur after decompression (Fig. 1).

Most of our patients experience an improvement of their symptoms in the following days. This could be explained by the preservation of the nerve structures without Wallerian-degeneration and per se lack of pain or neurological deficits with an improvement in symptoms during the days following surgery by this early recurrent Lumbar Disc Herniation [9–14].

The main limitation in our study is the retrospective data collection with its inherent bias, typical from this study design, with possible inconsistences in documentation. Furthermore it is not possible to differentiate with the data in the register, whether the cause for this early recurrent Lumbar Disc Herniation is remaining intervertebral disc tissue or a true recurrence.

## Conclusion

With help from the The German Spine Society Register we found a frequence of 1.2% in early recurrent LDH. Hence the German Spine Society Register seems to have a reliable data collection system that can perform multicentre data analysis. This data supports similar epidemiological studies to come to consistent values. The databases from this Register could be used in the future for various purposes, such as the evaluation of multicentre surgical techniques, results from patients with various surgical procedures and basic research in spinal surgery.
